# Increasing the Mechanical Strength and Corrosion Resistance of Aluminum Alloy 7075 via Hydrostatic Extrusion and Aging

**DOI:** 10.3390/ma15134577

**Published:** 2022-06-29

**Authors:** Marta Orłowska, Ewa Ura-Bińczyk, Lucjan Śnieżek, Paweł Skudniewski, Mariusz Kulczyk, Bogusława Adamczyk-Cieślak, Jarosław Mizera

**Affiliations:** 1 Faculty of Mechanical Engineering, Military University of Technology, gen. S. Kaliskiego 2, 00-908 Warsaw, Poland; lucjan.sniezek@wat.edu.pl; 2 Faculty of Materials Science and Engineering, Warsaw University of Technology, Wołoska 141, 02-507 Warsaw, Poland; ewa.ura@pw.edu.pl (E.U.-B.); pskudniewski@gmail.com (P.S.); boguslawa.cieslak@pw.edu.pl (B.A.-C.); jaroslaw.mizera@pw.edu.pl (J.M.); 3 Institute of High Pressure Physics, Polish Academy of Sciences, Sokolowska 29/37 St., 01-142 Warsaw, Poland; mariusz.kulczyk@unipress.waw.pl

**Keywords:** hydrostatic extrusion, AA 7075, microstructure, mechanical properties, corrosion

## Abstract

The present study investigates the correlation between mechanical properties and resistance to corrosion of hydrostatically extruded aluminum alloy 7075. Supersaturated solid solutionized samples undergo a plastic deformation process, followed by both natural and artificial aging. Furthermore, two types of hydrostatic extrusion are applied to the samples: single-stepped and double-stepped. This process is shown to influence grain refinement and the precipitation process, resulting in changes in the electrochemical properties of the samples. Hydrostatic extrusion combined with aging is shown to cause an increase in mechanical strength ranging from 50 MPa to 135 MPa in comparison to coarse-grained sample subjected to T6 heat treatment. The highest value of tensile strength is obtained for a sample subjected to single-step hydrostatic extrusion followed by natural aging. This strength increase is caused by refinement of the microstructure, in addition to the small size and number of precipitates at the grain boundaries, which are coarsened by artificial aging. Hydrostatic extrusion is also shown to increase resistance to corrosion, with the T6-treated coarse-grained sample being most susceptible to corrosion attack.

## 1. Introduction

Composed of Al-Zn-Mg(-Cu), 7xxx aluminum alloys (AAs) are of high interest to the aerospace industry due to their unique combination of light weight, high strength, and high resistance to corrosion [1]. These properties are superior to those of other AAs [2]. The complex microstructure of such materials reveals substantial potential for further improvement of its properties and manufacturing applications [3,4]. These AAs are age-hardenable, and therefore the precipitates are the primary strengthening factor. Additionally, the complexity of the microstructure is seen in numerous intermetallic particles with varying chemical compositions [5]. The application of an additional strengthening mechanism, such as grain refinement, has been shown to improve the properties of the material [6]. A group of methods known as severe plastic deformation (SPD) processes [7] can be used to refine the microstructure. SPD techniques are characterized by the application of a large strain, which introduces structural defects to the microstructure. It has been shown that SPD processing, applied to AA 7075 [8] via high-pressure torsion and equal channel angular pressing (ECAP), significantly reduced grain size to 310 nm. Correspondingly, a considerable increase in microhardness was obtained: from approximately 100 HV to 140 HV following ECAP alone, and to 250 HV after the combination of the two processes. Further research used ECAP to refine the microstructure of AA 7075 [9]. Four passes of the process (ε = 4), resulting in an average grain size of approximately 500 nm, increased the microhardness of a coarse-grained (CG) sample from 68 HV to 143 HV, and the tensile strength from 230 MPa to 440 MPa. The proportion of deformation determines the final grain size, fraction of high-angle grain boundaries (HAGBs), and dislocation density. This influences mechanical properties, as such structural defects being effective barriers for moving dislocations. Grain refinement also influences other properties, such as electrical conductivity [10], magnetic properties, or corrosion resistance [11]. In the case of the latter, the influence of microstructure refinement is ambiguous, and depends on parameters such as environmental conditions and the examined material. In the case of pure aluminum, as grain size decreases, the corrosion rate also decreases [12]. This correlation arises due to the following phenomenon—the refined microstructure has more reactive surfaces with respect to oxide formation and film ion conduction. From the other side, a higher number of grain boundaries can accelerate corrosion, as these are favorable paths of corrosion propagation [13]. Therefore, the deformation process influences the corrosion resistance, as it changes the characteristics of grain boundaries. The important aspect in this case is a value of the equivalent strain—ε. In SPD processing, it directly influences the grain refinement. In, e.g., the ECAP process, the value of ε can be estimated based on the relationship between the intersection angles of the channel in a die. This is described in [14]. In simplified terms, it can be assumed that in the case of a die with two channels with equal cross-section and with the intersecting channel angles equal to 90°, the outer arc of curvature equals 0°, and during one pass the ε ≈ 1.

**Figure 1:**
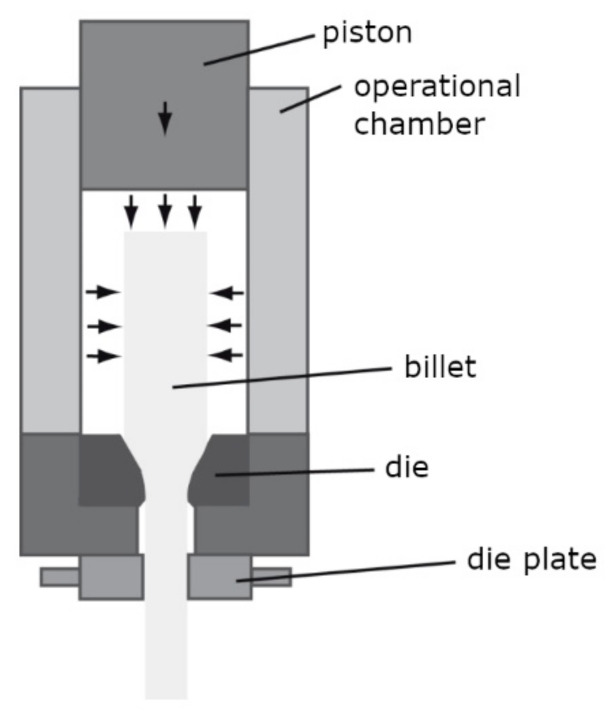
The scheme of hydrostatic extrusion process.

Hydrostatic extrusion (HE) [15] is an SPD method based on pressing a billet in a chamber using a fluid. A scheme of the process is presented in Figure 1. Due to the hydrostatic pressure, the size of the billet (e.g., the diameter in the case of rods) is reduced. The equivalent strain of hydrostatic extrusion is given by Equation (1) [16]:

(1) $$\varepsilon =2\ln\left(\frac{\;d\;\prime }{\;d\;\prime \prime }\right)$$

where d′ is the initial diameter of the rod and d″ is the exit diameter.

Unlike other SPD methods, such an approach can both obtain products of substantial length and also reduce strain, which is needed to refine the microstructure. However, due to the nature of the process, anisotropy is introduced to the microstructure, as grains are significantly elongated along the extrusion direction. Hence, mechanical anisotropy is observed [17]. Previous work has investigated the application of HE to 7xxx AAs. Lewandowska et al. [18] showed that applying HE to AA 7475 influenced the precipitation process, as an elevated number of nucleation sites caused a larger number of precipitates along boundaries than within grain interiors. Furthermore, the sizes of such precipitates were reduced. As a result, precipitate strengthening was reduced, as precipitates within grains interiors more strongly inhibit the movement of dislocations compared to those at grain boundaries. The results further revealed that appropriate heat treatment following HE can improve mechanical strength [6] due to the combination of grain boundaries and precipitation strengthening. Moreover, the resistance to pitting corrosion of AA 7475 samples that underwent HE was improved following aging [19]. Such an improvement is caused by the coarsening of stable intergranular MgZn_2_ precipitates and the formation of metastable MgZn_2_ in grain interiors during aging. As a result, corrosion resistance improved as the open circuit potential (E_OCP_) was shifted to more noble values and the corrosion current density (i_corr_) was reduced.

The literature study suggests there is high potential for 7xxx AAs to be endowed with substantial mechanical strength and superior corrosion resistance via appropriate thermo-mechanical treatment. Previous works have only investigated these two properties individually. Therefore, this paper adopts a more comprehensive approach, and attempts to correlate appropriate heat treatment of 7xxx AAs that have undergone HE with the development of high mechanical strength and high resistance to corrosion in an aggressive environment. This work is a continuation of our previous work, where the influence of heat treatment on the single-stepped hydrostatically extruded AA7075 was investigated [20].

## 2. Materials and Methods

AA 7075 was selected as the material under investigation. The exact chemical composition given by a producer is shown in Table 1. The material was delivered in the form of hot extruded bars with a diameter of 20 mm. The bars were supersaturated solid solutionized by annealing at 520 °C for 2 h, and then water-cooled. The billets were then plastically deformed via HE. Two HE approaches were chosen. In the single-stepped approach, the material was deformed in a single step with a reduction in diameter to 10 mm (ϕ10, ε = 1.41). In the multi-stepped approach, deformation consisted of two steps—an initial reduction to 12 mm, followed by a reduction to 8 mm (ϕ8). This approach produced an equivalent strain of Ɛ = 1.85.

The aging process was performed with the parameters shown in Table 2. The CG T6 sample was initially subjected to single-step HE, and then, to coarsen the grains, heat treated by supersaturated solid solutionizing and aged to a T6 state. In the case of the ultra-fine-grained (UFG) samples, two temperatures were examined, 100 °C and 120 °C, for 24 h. Additionally, samples after natural aging were investigated. Furthermore, retrogression and reaging (RRA) processes were applied to the ϕ10 UFG sample.

The sample microstructure was characterized using a Hitachi Su70 scanning electron microscope (SEM) with electron backscatter diffraction (EBSD) detector, and hence produced orientation maps (OIMs) and investigated average grain size, the fraction of HAGBs and low-angle grain boundaries (LAGBs), and texture. Pole figures (PFs) were generated during the analysis of the latter. Energy-dispersive X-ray spectroscopy (EDS) was used to determine the chemical composition of the intermetallic particles. A more detailed characterization of the microstructure was performed using a JEOL JEM 1200 transmission electron microscope (TEM). Samples that underwent microstructure characterization were electropolished using a Struers Tenupol-5 device using standard electrolyte A2. Moreover, X-ray diffraction (XRD) measurements were conducted with a use of Bruker D8 Advance diffractometer with Cu Kα radiation. The 2ϕ range of the XRD measurements was 25° to 90°.

The mechanical properties of the samples were investigated via static tensile tests and microhardness measurements. In the first case, flat mini-samples with a cross section of 0.6 × 0.8 mm and a gauge length of 5 mm were used [21,22]. Tests were performed at a strain rate of 10^−3^ s^−1^ with the use of the digital image correlation for the measurement of the strain. The tests produced quantitative results for average values of ultimate tensile strength (UTS), yield strength (YS), and elongation at break (E_b_). Samples were cut longitudinally to the extrusion direction. For each state, three samples were tested. Microhardness measurements were conducted using a Vicker’s method with a load of 200 g (HV0.2). Five measurements per sample were conducted.

The corrosion resistance of the samples was investigated via electrochemical experiments in quiescent 0.1M NaCl solution. The samples underwent cyclic polarization in potentiodynamic (PP) mode. Before each test, each specimen was immersed within a prepared electrolyte for 10 min to stabilize the rest potential. The tests were performed using a NOVA AutoLab PGSTAT302N potentiostat/galvanostat in three electrode configurations, with the sample acting as the working electrode, a silver chloride reference electrode, and a platinum sheet counter electrode. A 20 mm^2^ area on the surface of each sample was examined. Corrosion tests were conducted on the surfaces perpendicular to the HE direction. PP was performed with a scan rate of 1 mV/s starting from −0.05 mV relative to E_OCP_, and reversed when the current reached 0.25 mA. PP measurements were repeated three times to ensure reproducibility of the results. Average values of corrosion potential (E_corr_), corrosion current density (i_corr_), and repassivation potential (E_rep_) were obtained. All tests were performed at room temperature.

## 3. Results

### 3.1. Microstructure Evolution

#### 3.1.1. SEM

**Figure 2:**
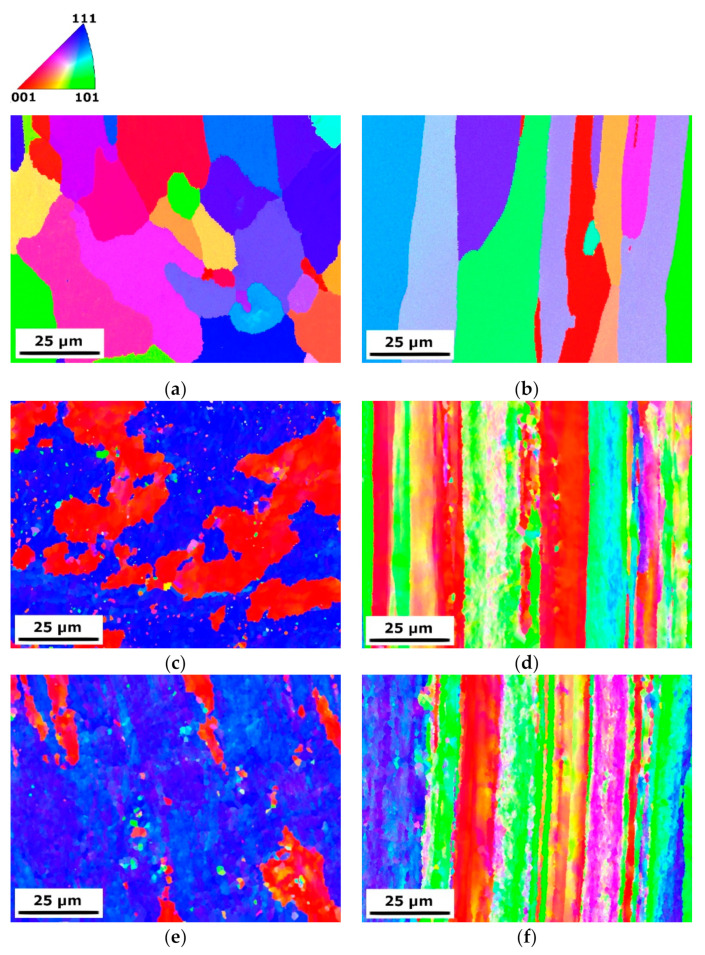
OIMs of the: (**a**) CG T6 transverse plane, (**b**) CG T6 longitudinal plane, (**c**) HE10 transverse plane, (**d**) HE10 longitudinal plane, (**e**) HE8 transverse plane and (**f**) HE8 longitudinal plane.

**Figure 3:**
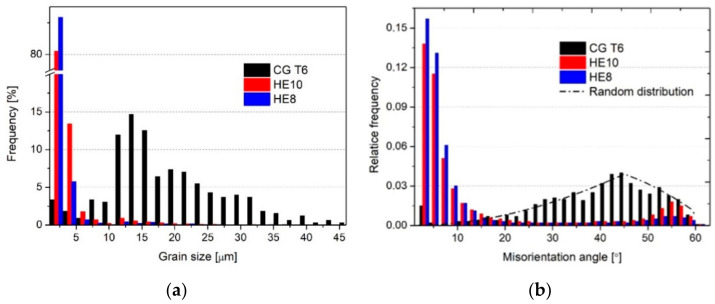
The distribution of: (**a**) grain size and (**b**) misorientation angle on the transverse plane of the CG T6, HE10, and HE8 samples.

Figure 2 shows OIMs of CG T6, HE10 and HE8 samples on two planes: transverse and longitudinal to the extrusion direction. The distribution of grain size and the grain boundary misorientation angles are presented in Figure 3. The average grain size for the CG T6 sample is approximately 17 µm on both planes. The grain shape differs, with equiaxial grains on the transverse plane and elongated grains on the longitudinal plane. Both planes contain more than 90% of HAGBs. Following HE, substantial grain refinement can be observed, primarily indicated by a considerable increase in the number of LAGBs. For both deformed samples HE10 and HE8, the microstructure along the transverse plane displays equiaxial grains with a considerable fraction of LAGBs, which are randomly distributed to create a network of subgrains within the grains, which are surrounded by HAGBs. The average grain/subgrain size is decreased to 1.5 µm and 1.0 µm for HE10 and HE8, respectively. Those samples also have HAGB fractions of 24% and 15%, respectively. As seen in Figure 3, the most frequent grain sizes for the HE8 sample are below 3 µm. In addition, the majority of grain boundary misorientation angles are below 10°. These findings indicate that further grain refinement was primarily due to the formation of subgrains separated by LAGBs. A substantial difference in the microstructure can be observed on the longitudinal plane. Fibrous grains are formed, manifesting as highly elongated shapes. Straight HAGBs are aligned along the extrusion direction. Within the fibrous grains, a network of LAGBs can be observed. The average grain size is larger than that on the transverse plane, although the distance between HAGBs is smaller.

**Figure 4:**
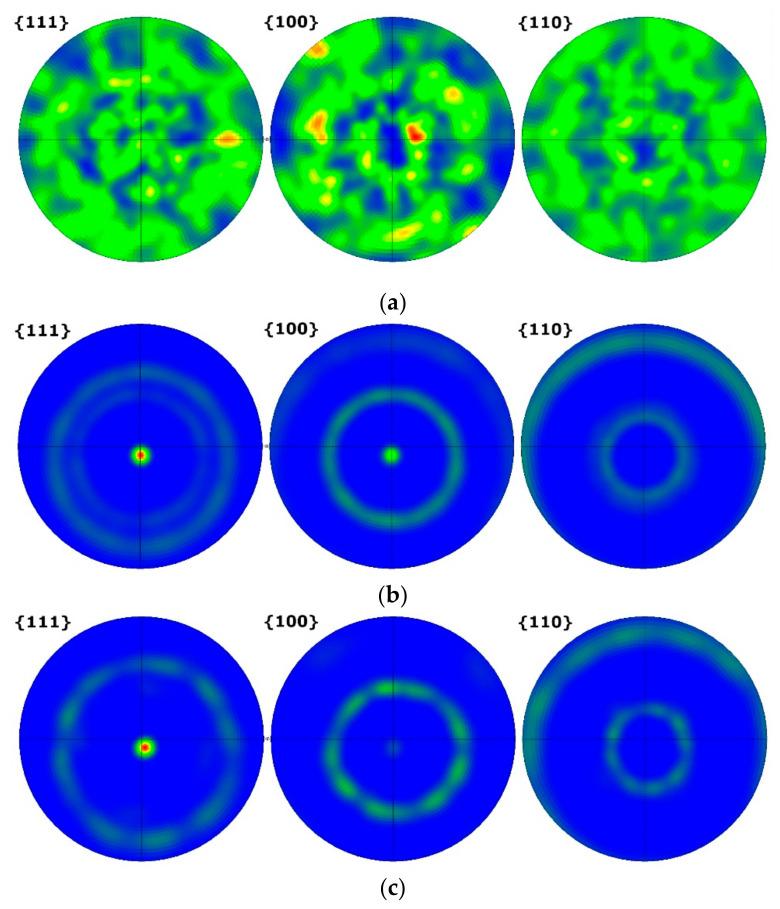
PFs of the: (**a**) CG T6, (**b**) HE10, and (**c**) HE8 samples.

Figure 4 shows PFs for the transverse plane of the samples. The CG T6 sample exhibits random grain orientations without a distinct texture. Following HE, the HE10 and HE8 samples display substantially changed grain orientations, with <111> and <001> dominant for HE10 and <111> dominant for HE8. Moreover, a higher intensity can be observed for the HE8 sample, corresponding with stronger deformation.

**Figure 5:**
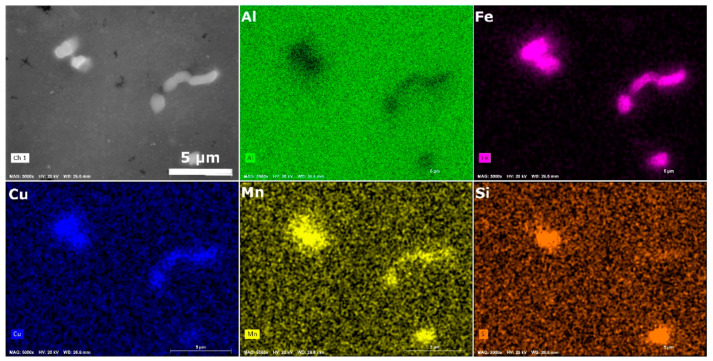
SEM/EDS analysis of the intermetallic particles.

Figure 5 presents the results of EDS analysis, used to characterize the intermetallic particles within the microstructure. A diverse range of particles can be observed, with the majority rich in Cu and Fe, in addition to Mn and Si. In terms of electrochemical potential, both Fe and Cu are nobler than the Al matrix [23]. This influences resistance to pitting corrosion, as pit nucleation is initiated in the vicinity of intermetallic particles. In terms of mechanical properties, such particles do not contribute to the increase in strength of the material.

**Figure 6:**
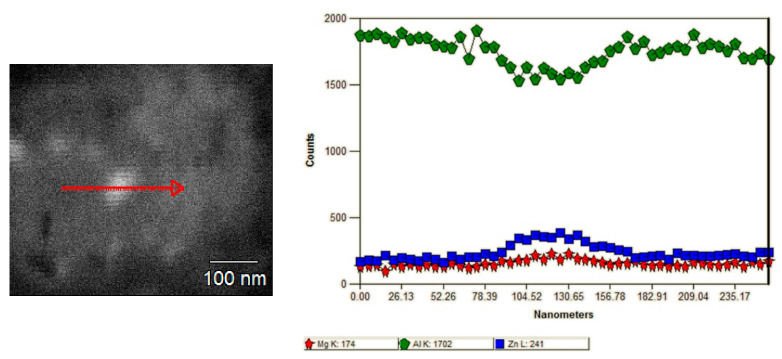
SEM/EDS analysis of the second-phase precipitate.

Moreover, for the characterization of second-phase precipitates, the EDS analysis was also performed. The results are shown in Figure 6, where a line scan of the exemplary precipitate is presented. It can be seen that the precipitate is rich Zn and Mg. Both η and η′ precipitate are rich in these elements. For AA7075, these two precipitates are the major precipitates in the microstructure, as can be seen in numerous papers, e.g., [24,25,26,27].

#### 3.1.2. TEM

**Figure 7:**
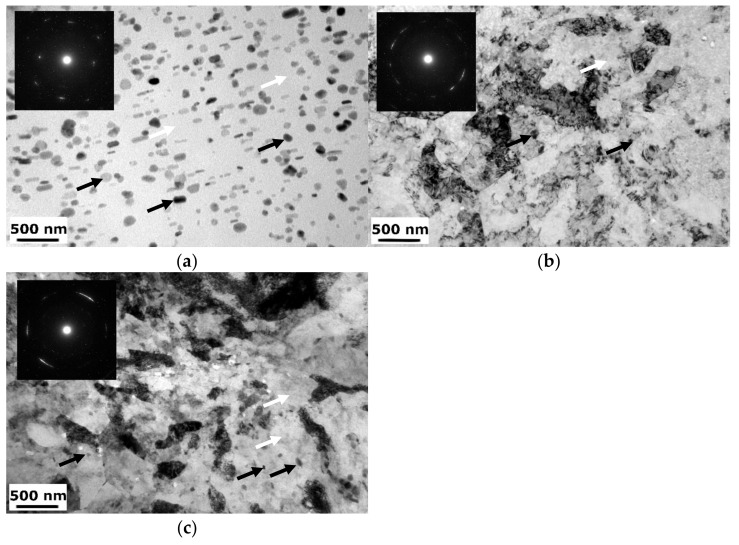
TEM micrographs of the: (**a**) CG T6, (**b**) HE10 HT2, and (**c**) HE8 HT2 samples, black arrows indicate η precipitates, white arrows—η′ precipitates.

A detailed characterization of the microstructure was performed using TEM. Figure 7 presents example micrographs of the transverse plane of the undeformed and deformed samples further aged at 120 °C/24 h. The micrographs reveal differences between the samples in terms of plastic deformation and heat treatment. Samples display different grain size and density of dislocations. The CG T6 sample is characterized by a coarse-grained microstructure with a high density of η precipitates within the grain interiors, with additional η′ precipitates visible as small dots within the matrix. Similar observation of the microstructure components were found in, e.g., [24,28,29] The microstructures of the HE10 HT2 and HE8 HT2 samples are substantially deformed, which can be seen by the grain refinement, but also feature significant increases in dislocation density. Moreover, it is seen in selected area diffraction images, which are inside the TEM micrographs. For samples after hydrostatic extrusion, the diffractions are close to rings, which indicates a higher number of orientations in comparison to CG T6 sample. In addition, the size of η precipitates is reduced in comparison to the CG T6 sample.

**Figure 8:**
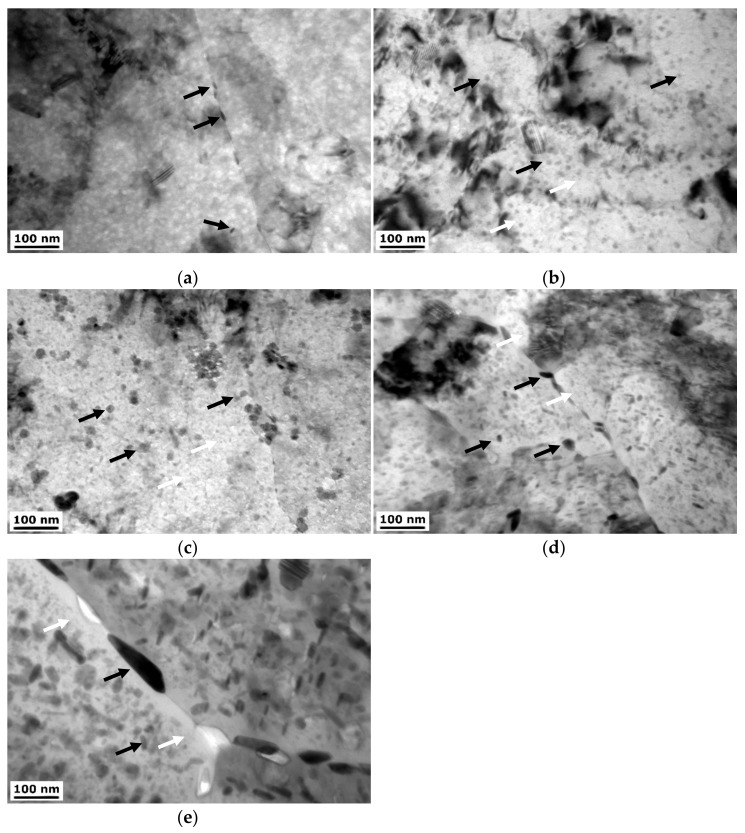
TEM micrographs of the: (**a**) HE10, (**b**) HE10 HT1, (**c**) HE10 HT2, (**d**) HE10 RRA1, and (**e**) HE10 RRA2 samples, black arrows indicate η precipitates, white arrows—η′ precipitates.

The TEM micrographs were further analyzed at higher magnification. Figure 8 shows micrographs of the ϕ10 samples. The size and number of the η precipitates are substantially reduced within the naturally aged sample in comparison to the CG T6 sample (Figure 7). Artificial aging leads to the intensification of precipitation process, and the number and size of precipitates increase. Precipitates are observed at both grain boundaries but also grain interiors. With increasing the aging temperature, the size of the η precipitates increases. What can be noticed is that RRA heat treatment led to over-aging of the material. This can be seen by the significant coarsening of η precipitates, most likely as a cost of η′ precipitates. A higher number of the precipitates can be observed at the grain boundaries. Moreover, precipitate-free zones (PFZ) next to the grain boundaries can be observed with a thickness up to 30 nm.

**Figure 9:**
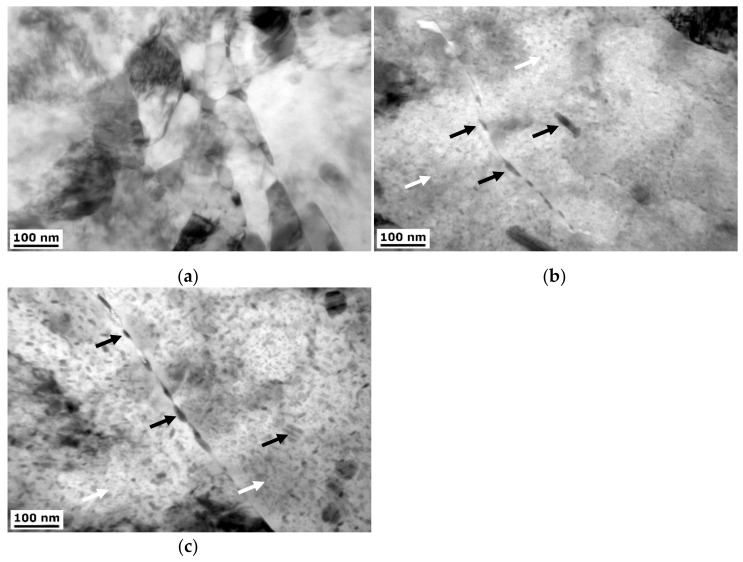
TEM micrographs of the: (**a**) HE8, (**b**) HE8 HT1, and (**c**) HE8 HT2 samples, black arrows indicate η precipitates, white arrows—η′ precipitates.

Figure 9 shows TEM micrographs of the ϕ8 samples. A similar phenomenon can be observed as for the ϕ10 samples. Again, as the aging temperature increases, the size of the precipitates increases. This effect is stronger for the ϕ8 samples than for the ϕ10 samples, with more pronounced precipitate growth at the same aging temperature. Furthermore, PFZs are already observed at an aging temperature of 120 °C, indicating over-aging. The HE8 sample displays more pronounced grain refinement in comparison to the HE10 sample. Grains of nanometer size (below 100 nm) can already be observed, which was not the case for the HE10 sample. Artificial aging resulted in a precipitation process. In addition, the number of grain boundary precipitates is higher in comparison to samples with lower deformation rate.

#### 3.1.3. XRD

**Figure 10:**
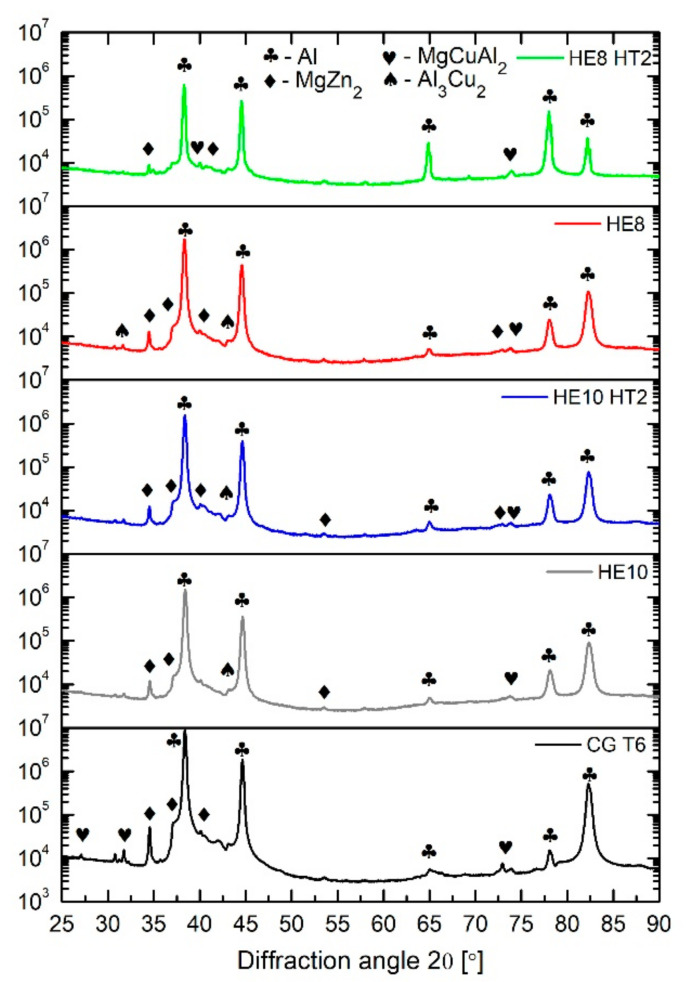
XRD analysis of the CG T6, HE10, HE10 HT2, HE8 and HE8 HT2 samples (PDF no. of the phases identified: PDF 00-004-0787—Al, PDF 01-073-5874—MgCuAl_2_, PDF 00-034-0457—MgZn_2_, PDF 01-071-5716—Al_3_Cu_2_).

XRD analysis was performed to confirm TEM observations. Figure 10 shows results of the analysis for samples CG T6, HE10, HE10 HT2, HE8, and HE8 HT2. For all the samples, the same phases have been identified. For each sample, the most prominent phases are Al solid solution, η precipitates (labeled MgZn_2_), MgCuAl_2_, and Al_3_Cu_2_. In addition, η′ precipitates were detected, but their very small size resulted in diminished XRD peaks. Similar observations were found in work [30], where XRD measurements of AA7075 revealed peaks for MgZn_2_ and Al_2_Cu; however, the microstructure observations show the presence of MgZn_2_. As in the present study, this indicates that these precipitates constitute the majority of the microstructure.

### 3.2. Mechanical Properties

#### 3.2.1. Microhardness Measurements

**Figure 11:**
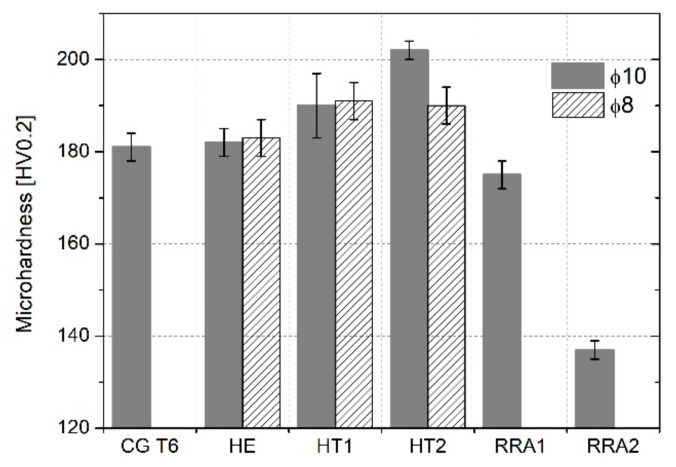
Microhardness results of the samples.

Figure 11 presents a graph of the microhardness measurement results. The CG T6 sample has an average hardness of 181 HV0.2. Following HE and natural aging, the microhardness changes are insignificant. The HT1 and HT2 treatments improved the microhardness of both samples. However, in the case of the ϕ8 samples, microhardness saturation was already observed for HT1, and a further increase in aging temperature caused a slight decrease in microhardness. In the case of the ϕ10 samples, the highest microhardness value of 202 HV0.2 was obtained for the HE10 HT2 sample. RRA treatment substantially decreased microhardness below that of the CG T6 sample. Based on these results, samples RRA1 and RRA2 were not investigated further.

#### 3.2.2. Tensile Tests

**Figure 12:**
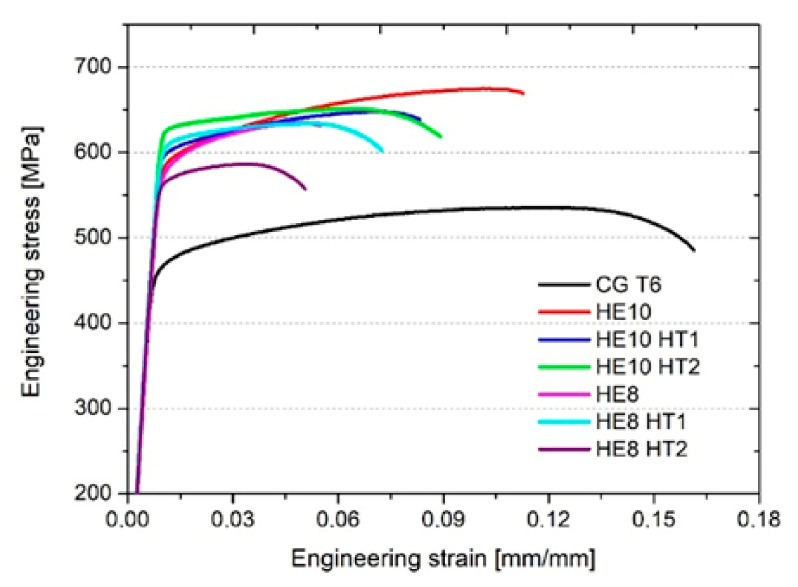
Representative engineering stress–strain curves for the reference CG T6 sample and samples after hydrostatic extrusion with subsequent natural aging (HE10 and HE8) and artificial aging (HT1 and HT2).

The representative stress–strain curves obtained by the tensile tests are shown in Figure 12. Table 3 presents the quantitative results. The samples were tested in the direction longitudinal to the extrusion direction. The CG T6 sample displays the lowest UTS value of about 540 MPa. All the samples that underwent HE show an improvement in UTS and YS, but a reduction in E_b_. The increase in UTS, depending on a sample, equals from 50 MPa to 135 MPa, indicating a significant improvement. The tensile strength of the plastically deformed samples is dependent on the aging temperature. For the ϕ10 samples, the highest results are observed for the naturally aged sample, with a UTS and E_b_ of 674 MPa and 17%, respectively. Both HT1 and HT2 show a slight decrease in YS, UTS, and E_b_. Among the ϕ8 samples, the highest strength of 625 MPa was obtained for the HE8 HT1 samples. However, it has to be emphasized that for the ϕ8 samples, the variations in the results is considerable, indicating the inhomogeneity of the microstructure.

### 3.3. Electrochemical Properties

**Figure 13:**
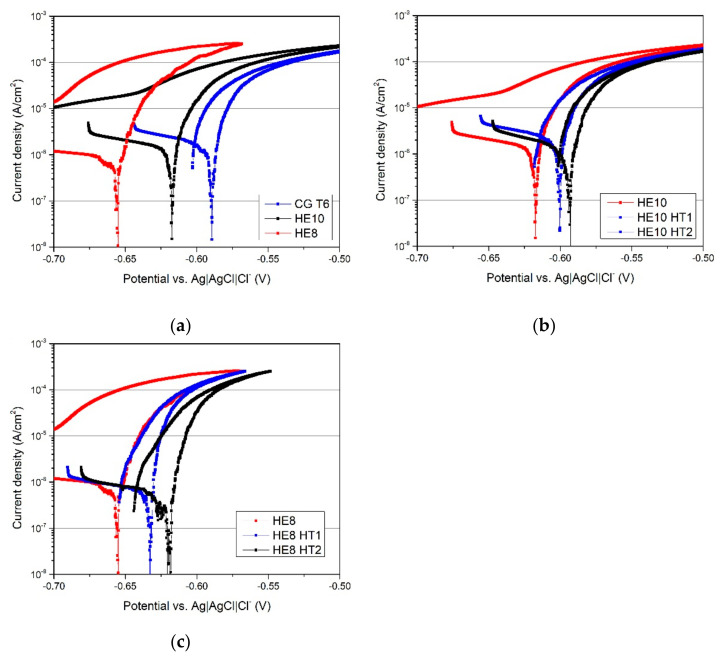
Results of PP tests in 0.1M NaCl, for the (**a**) CG T6, HE10, and HE8 samples; (**b**) the HE10 sample before and after aging; and (**c**) HE8 sample after aging.

**Figure 14:**
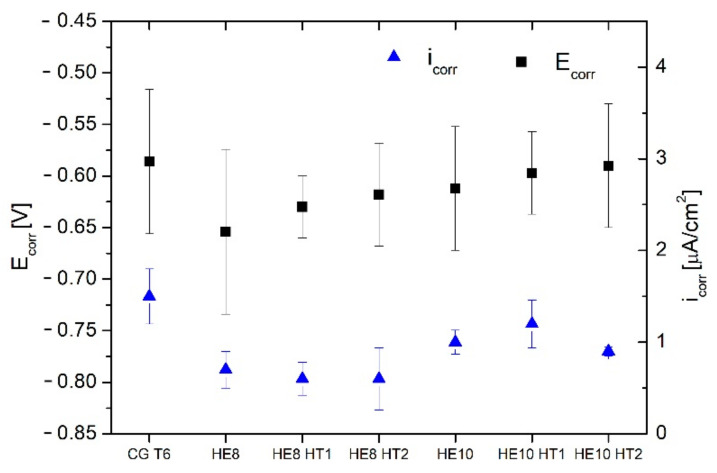
The electrochemical parameters E_corr_, E_rep_ (left Y axis), and i_corr_ (right Y axis) for each sample.

Figure 13 shows the curves generated from the PP tests, and Figure 14 shows the average electrochemical parameters for each sample. The polarization curves for the HE and CG T6 samples are generally similar in shape, with the principal difference being the size of hysteresis loop (Figure 13a). As deformation increases, the hysteresis loop becomes larger. This indicates that the repassivation process is slower for the refined samples, and consequently suggests that the morphology of corrosion attacks may differ. The cathodic current density during the forward scan substantially decreases with increasing plastic deformation of the microstructure (Figure 13a). The corrosion potentials (E_corr_) of the HE samples are lower than those of the CP T6 sample (Figure 14). The anodic behavior is dominated by active dissolution, as characterized by the lack of a passive region and an abrupt increase in current density above the corrosion potential.

Following artificial aging, the repassivation rates of both refined samples increase, as the hysteresis loop defined by the reverse scan curve becomes smaller (Figure 13b,c). This suggests a change in the morphology of the corrosion attack [31]. In the cathodic branch, the current density of the HE8 sample remains unaffected by aging, while that of the HE10 sample increases slightly. As aging temperature increases, E_corr_ shifts to more noble values. Most notably, the corrosion currents (i_corr_) of all samples which are either refined or both aged and refined are lower than that of the CG T6 sample, indicating a slight improvement in corrosion resistance. Note that the differences in E_corr_ and i_corr_ are small, and the major dissimilarity in corrosion resistance lies in the size of the hysteresis loop, and hence the morphology of the corrosion attack.

**Figure 15:**
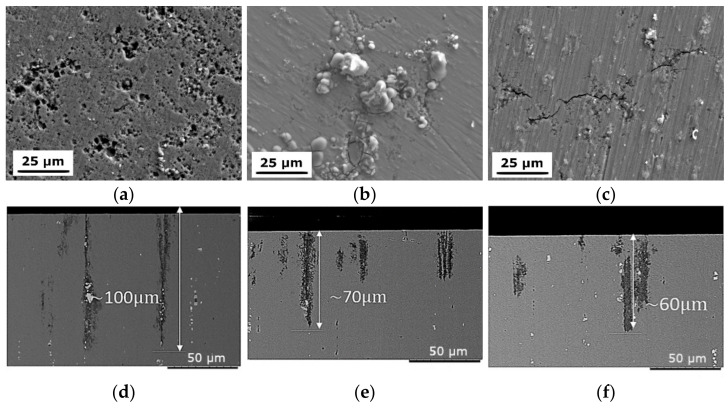
SEM micrographs of the surfaces of: (**a**) CG T6, (**b**) HE10, (**c**) HE8 and cross-sections of (**d**) CG T6, (**e**) HE10, (**f**) HE8 samples. 50 µm.

Following PP testing, the microstructure was investigated further. Figure 15 shows SEM micrographs of the surfaces and cross-sections of the CG T6, HE10, and HE8 samples. Numerous pits can be observed on the CG T6 sample, where the whole examined surface was covered with them. For the samples that underwent HE, the corrosion attack is limited and more localized. For the HE8 sample, the corrosion attack on the surface looks like an intergranular, where the grain boundaries are more susceptible to the attack. Moreover, corrosion products can be observed on the surfaces. The cross-sections of all three samples display corrosion attack, which can be considered as combined intergranulars with pitting. Pits initiate in the vicinity of the intermetallic particles and further propagate along the extrusion direction, and correlate with highly elongated grains. Dissolution occurs along grain boundaries. As such, the dissolute networks of the HE10 and HE8 samples are more developed, as those samples possess a higher density of grain boundaries. In the case of the length of the corrosion attack, it varied between the samples. It was the most significant in the case of the CG T6 sample and equaled up to 100 µm. For the refined samples, this value was reduced and did not exceed 70 µm. Differences between HE10 and HE8 samples are negligible.

## 4. Discussion

### 4.1. The Influence of Microstructure Evolution on Mechanical Properties

Plastic deformation via HE in the present study with the application of low values of strain [32] led to the formation of substructures, within which the majority of grain boundaries were LAGBs. Higher strain values would be required to generate HAGBs by rotating the subgrains over 15°. The evolution of grain boundary angles during HE has been analyzed previously [33]. The misorientation angles are greatest when between grains oriented in the <111> and <001> directions. When the boundary lies between grains of similar orientations, then the misorientation angle in substantially lower. However, as the strain increases during deformation, the misorientation angles of such boundaries are subject to the largest increase. The grain refinement of the ϕ8 samples was slightly greater than that of the ϕ10 samples. This was reflected in an increased number of LAGBs, which resulted in more pronounced substructure formation. The grain orientation was somewhat invariant, with the majority of grains oriented along the <111> direction.

When compared to other SPD processes, such as ECAP or high-pressure torsion, HE produces grains of a distinctive shape. The grains are strongly elongated along the extrusion direction (longitudinal plane), while on the transverse plane the grains are equiaxial (see Figure 2). On the longitudinal plane the fibrous grains are defined by HAGBs, while their interiors feature networks of LAGBs. In terms of mechanical strength, the Hall–Petch equation describes an increase in yield strength as grain size decreases [34,35]. However, strength is also influenced by structure—namely, the grain boundary misorientation angle [36]. Therefore, the ϕ8 sample displayed no further increase in mechanical strength when compared to the ϕ10 sample, as the majority of grains were LAGBs. Such grains provide a weaker barrier to moving dislocations in comparison to HAGBs, and hence impart a lower contribution to strengthening the material [37]. Grain boundaries act as the primary strengthening mechanism in the case of plastic deformation; as the applied strain increases—creating HAGBs with smaller grain sizes—the mechanical strength increases. Accordingly, the results in this paper show that YS and UTS were significantly higher for the samples that underwent HE than the CG T6 sample. Note also that the increase in applied strain (from 1.41 to 1.85), which resulted in more substantial grain refinement, did not cause further improvement in mechanical strength. In addition to the misorientation angle between grains, precipitates are also a strengthening factor, which needs to be discussed.

In work [38], AA 7075 samples with both CG and UFG microstructure were aged. The results showed that, in equivalent conditions, the UFG sample obtained higher mechanical strength. However, precipitation strengthening was more impactful for the CG sample. Following T6 aging, the yield strength increment was 160% for the CG sample and only 26% for the UFG sample. The difference was caused by the size of the η and η′ phase precipitates within the grain interiors, as these precipitates pin dislocations. This is known as the Orowan mechanism. The size of the strengthening precipitates was less than 5 nm within the UFG sample; such precipitates within the CG sample were much larger, at approximately 60 nm. Further work obtained similar findings when examining AA 7475 following HE [18]. Grain boundaries and dislocations are favorable locations for precipitation nucleation; however, such precipitates do not contribute to the strengthening of the material. Therefore, to obtain a high mechanical strength, the number of boundary precipitates should be reduced. This phenomenon explains the smaller values of YS and UTS obtained for the ϕ8 samples in comparison with the ϕ10 samples. In the case of the latter one, due to reduced grain refinement resulting from lower applied strain, the volume fraction of both grain boundaries and boundary precipitates was smaller. The HE10 sample developed the highest UTS value due to an optimal correlation between the number of grain boundaries and natural aging, engendered by a slower precipitation process. Precipitation was accelerated by the artificial aging process, resulting in a higher number of boundary precipitates and therefore a decrease in mechanical strength. In terms of mechanical strength, the elevated number of grain boundaries was unfavorable for the ϕ8 samples, as they caused a higher number of boundary precipitates at the expense of interior precipitates, which act as strengthening factors.

The ϕ10 samples were additionally subjected to RRA. This approach was taken to obtain high mechanical strength [39] together with excellent corrosion resistance. Following heat treatment to a T6 state, the CG T6 sample was heated to retrogression temperature and aged for a short time. Following RRA treatment, the resultant microstructure was more thermodynamically stable, and consisted of a greater number of bigger precipitates when compared to those formed due to the T6 heat treatment only [40]. Previous work showed that RRA reduced the corrosion depth of AA 7075, while mechanical strength remained high if parameters were optimized [41]. Due to adiabatic heating, which momentarily causes the temperature to increase to approximately 100–150 °C, the HE process influences precipitation. Further work, using AA 6082, showed that the precipitation sequence changed during HE, creating an additional phase capable of nucleation, which was not present within a CG sample [42]. The precipitation process differs also because of the increased number of structural defects, such as grain boundaries and dislocations, on which nucleation can occur [18]. Hence, RRA treatment of the HE samples caused a substantial decrease in microhardness. Following HE, the microstructure is much more susceptible to the precipitation process. Precipitation both along the grain boundaries and within the grain interiors underwent significant coarsening. Due to the increased number of grain boundaries, the number of boundary precipitates also increased. Following RRA treatment, the growth of such precipitates caused a substantial decrease in microhardness, and the obtained results showed that in the case of UFG sample, the RRA treatment is not effective and leads to extensive over-aging.

### 4.2. The Evolution of Resistance to Corrosion

The microstructure evolution during HE with the following aging influences the electrochemical properties of the AA 7075, as presented in Figure 14. The CG T6 sample displayed the highest E_corr_ values. For the samples that underwent HE, these parameters were reduced. However, the value of i_corr_, which is directly correlated with corrosion rate, was reduced after HE process. The lowest values of i_corr_ were obtained for the ϕ8 samples, where the artificial aging resulted in further slight improvement. In the case of the ϕ10 samples, HE10 and HE HT12 displayed similar values of i_corr_, with the latter having increased values of E_corr_ and E_rep_. The quantitative results obtained from the electrochemical tests together with data from the literature are gathered in Table 4. The results for AA7075 varies in dependance of the corrosion environment—for higher concentration of NaCl, lower values of E_corr_ and higher values of i_corr_ are achieved. Nevertheless, the obtained results in a present study are within the results obtained in the literature. Upon investigation of the sample surfaces following PP testing, the CG T6 sample had the most pronounced susceptibility to corrosion attack. The corrosion attack was much more localized for the samples that underwent HE, with a limited number of pits observed on their surfaces. The entire examined area of the CG T6 sample was damaged, indicating that the corrosion was more uniform, rather than producing individual pits. However, upon examination of the cross-sections, all samples displayed pitting/intergranular corrosion. The pits were spread along the HE direction. A greater number of surface pits means a larger number of potential locations for further propagation of the corrosion into the material. The smaller number of pits observed for the samples that underwent HE may be caused by a denser passive layer. A more stable and integral passive layer has been observed for UFG materials in relation to the corrosion resistance of materials following SPD processing [13,43]. This phenomenon is caused by an elevated number of structural defects such as grain boundaries and dislocations, which are favorable locations for oxidation.

Although corrosion attacks were observed in the vicinity of intermetallic particles, such particles themselves remained untouched. This is due to the higher electrochemical potential of these particles, which primarily contain Fe and Cu, in comparison to the Al matrix [23,49]. Further dissolution of the material takes place via grain boundaries. Previous work showed that HAGBs provide preferable locations for the propagation of corrosion due to the presence of enhanced excess energy [13]. Further work found that AA 5182 was susceptible to intergranular corrosion due to β-phase precipitates at grain boundaries, and that LAGBs provide resistance to attack [50]. HAGBs provided a variable resistance to attack, dependent upon the grain boundary plane. This could be caused by the crystallography of β-phase precipitation, which determines whether precipitation along boundaries is continuous or discontinuous. For 7xxx AAs, intergranular corrosion was shown to be caused by the presence of PFZs [51], and susceptibility to intergranular corrosion was reduced by decreasing the sizes of the PFZs. Higher susceptibility to pitting corrosion is unfavorable as it can initiate cracking, as shown in [52], where authors investigated the transition of corrosion pits to cracks under fatigue for AA 7075. 

Naturally aged, fine-grained AA 7075 was less susceptible to the growth of stable pits compared to CG AA 7075 in [47]. This finding was attributed to the size of the intermetallic particles, which were larger for the CG sample. The potential difference between the cathodic particles and the matrix was higher in the CG sample than in the refined sample. This was caused by a higher content of alloying elements within particles, resulting in a greater driving force for pitting initiation and propagation in the CG sample. However, in this paper, the size of the intermetallic particles was constant across all samples. Therefore, the primary cause of the enhanced corrosion resistance of the deformed samples is grain refinement, which causes a better protective passive layer on the surface and therefore the number of corrosion nucleation sites is smaller in comparison to the CG T6 sample. Further propagation of the corrosion occurs along HAGB, and the observed corrosion damage is similar for the samples.

## 5. Conclusions

In this paper, AA 7075 was subjected to HE and aging. The microstructure evolution, mechanical properties, and resistance to corrosion were examined, and the results were compared to a CG T6 sample. From this, the following conclusions can be drawn:

- The HE process caused substantial grain refinement; however, the applied strain values of ε = 1.41 and ε = 1.85 resulted in the majority of grain boundaries being LAGBs;
- The samples that underwent HE displayed different precipitation phenomena—the increased number of grain boundaries resulted in a higher number of boundary precipitates, which do not contribute to an increase in mechanical strength;
- HE with an aging process caused an increase in tensile strength in a range of 50–135 MPa in comparison to CG T6 sample. The highest value of 675 MPa was obtained for singe-stepped HE with natural aging;
- Due to the increase in applied strain during HE, a lower aging temperature was required to develop high mechanical strength;
- The samples which underwent HE showed lower susceptibility to localized corrosion in comparison to the CG T6 sample as a result of the grain refinement and higher fraction of LAGBs, which are resistant to corrosion attack.

## Figures and Tables

**Table 1:** The chemical composition of AA 7075.

Element	Zn	Mg	Cu	Cr	Ti	Si	Fe	Mn	Al
Content (wt. %)	5.70	2.40	1.50	0.19	0.04	0.09	0.23	0.06	balanced

**Table 2:** Sample designations with respect to the applied heat treatments.

	Naturally Aged/180 Days	100 °C/24 h	120 °C/24 h (T6)	RRA 120 °C/24 h + 200 °C/40 min + 120 °C/24 h	RRA 120 °C/24 h + 240 °C/40 min + 120 °C/24 h
ϕ10 CG	-	-	CG T6	-	-
ϕ10 UFG	HE10	HE10 HT1	HE10 HT2	HE10 RRA1	HE10 RRA2
ϕ8 UFG	HE8	HE8 HT1	HE8 HT2	-	-

**Table 3:** Results obtained from the tensile tests.

Sample	YS [MPa]	UTS [MPa]	E_b_ [%]
CG T6	463 ± 4	539 ± 3	16.9 ± 1.4
HE10	582 ± 2	674 ± 2	11.0 ± 0.2
HE10 HT1	603 ± 11	652 ± 8	8.8 ± 0.5
HE10 HT2	608 ± 22	639 ± 19	9.3 ± 0.2
HE8	552 ± 33	608 ± 53	4.7 ± 2.8
HE8 HT1	588 ± 24	625 ± 18	7.5 ± 1.9
HE8 HT2	563 ± 16	590 ± 11	5.4 ± 1.1

**Table 4:** Results of electrochemical properties with comparison to the literature data.

Sample	E_corr_ [mV]	i_corr_ [µA/cm^2^]	Environment	Ref.
CG T6	−585	1.5		Present study
HE10	−612	1.0		
HE10 HT1	−597	1.2		
HE10 HT2	−590	0.9	0.1 M NaCl	
HE8	−654	0.7		
HE8 HT1	−630	0.6		
HE8 HT2	−618	0.6		
AA7075 with different tempers	−800 to −770	-	3.5% NaCl	[44]
AA7075	−815	4.01	3.5% NaCl	[45]
	−853	4.3	1 M NaCl	
	−945	6.76	2 M NaCl	
	−1038	9.58	3 M NaCl	
AA7075 CG	−1165	76.2	3.5% NaCl	[26]
AA7075 after rolling	−1162 to −1088	5.4 to 55.9		
AA7075	−704	71.5	3.5% NaCl	[46]
AA7075	−553 to −497	0.1 to o.5	0.001 M NaCl	[47]
AA7075 welds	−1350 to −750	-	3.5% NaCl	[48]

## Data Availability

The data presented in this study are available on request from the corresponding author.
